# Inattentive Delirium vs. Disorganized Thinking: A New Axis to Subcategorize PACU Delirium

**DOI:** 10.3389/fnsys.2018.00022

**Published:** 2018-05-23

**Authors:** Darren F. Hight, Jamie Sleigh, Joel D. Winders, Logan J. Voss, Amy L. Gaskell, Amy D. Rodriguez, Paul S. García

**Affiliations:** ^1^Department of Anaesthesiology, Waikato Clinical Campus, University of Auckland, Hamilton, New Zealand; ^2^Waikato District Health Board, Hamilton, New Zealand; ^3^Research Division, Atlanta VA Medical Center, Atlanta, GA, United States; ^4^Department of Anesthesiology, Emory University School of Medicine, Atlanta, GA, United States

**Keywords:** general anesthesia, post anesthetic care unit (PACU), post-operative delirium, CAM-ICU, disordered thinking, inattention, sedation

## Abstract

**Background:** Assessment of patients for delirium in the Post Anesthesia Care Unit (PACU) is confounded by the residual effects of the varied anesthetic and analgesic regimens employed during surgery and by the physiological consequences of surgery such as pain. Nevertheless, delirium diagnosed at this early stage has been associated with adverse clinical outcomes. The last decade has seen the emergence of the confusion assessment method-intensive care unit (CAM-ICU) score as a quick practical method of detecting delirium in clinical situations. Nonetheless, this tool has not been specifically designed for use in this immediate postoperative setting.

**Methods:** Patients enrolled in a larger observational study were administered the CAM-ICU delirium screening tool 15 min after the latter of return of responsiveness to command or arrival in the post-anesthesia care unit. Numerical pain rating scores were also recorded. In addition, we reviewed additional behavioral observations suggestive of disordered thinking, such as hallucinations, a non-reactive eyes-open state, or an inability to state a pain score.

**Results:** Two-hundred and twenty-nine patients underwent CAM-ICU testing in PACU. 33 patients (14%) were diagnosed with delirium according to CAM-ICU criteria; 25 of these were inattentive with low arousal, seven were inattentive with high arousal, and one was inattentive and calm and with disordered thinking. Using our extended criteria an additional eleven patients showed signs of disordered thinking. CAM-ICU delirium was associated with increased length of operation (*p* = 0.028), but a positive CAM-PACU designation was associated with both increased operation length and age (*p* = 0.003 and 0.010 respectively). Two of the CAM-ICU positive patients with inattention and high arousal reported high pain scores and were not classified as CAM-PACU positive.

**Conclusion:** Disordered thinking is correlated with older patients and longer operations. The sensitivity of the existing CAM-ICU score in diagnosing delirium or disordered thinking in PACU patients is improved by the inclusion of a few extra criteria, namely: patients having perceptual hallucinations, in an unreactive eyes-open state, or who cannot state a pain score. We present this alternative screening tool for use in the post-anesthetic period, which we have named CAM-PACU.

## Introduction

Delirium is defined as an acute (and often fluctuating) organic brain disorder of both attention and cognition characterized by: clouding of consciousness, reduced ability to focus and sustain attention on the environment, and cognitive and perceptual disturbances (European Delirium Association, 2014; Hayhurst et al., 2016). Delirium can be sub-classified as hyperactive, hypoactive and mixed (Meagher et al., 2012; Mashour et al., 2015) and postoperative delirium is associated with a variety of poor clinical outcomes (Saczynski et al., 2012) some of which can be prolonged and extreme (Whalin et al., 2015). The Diagnostic and Statistical Manual of Mental Disorders, Fifth Edition (DSM-5; Association of American Psychiatric Association, 2013) is considered to be the gold standard for definition and diagnosis of delirium (Hayhurst et al., 2016). A number of instruments have been validated for screening delirium (Hayhurst et al., 2016); one of the most popular of these instruments, the Confusion Assessment Method for the Intensive Care Unit (CAM-ICU; (Ely et al., 2001) was developed to screen for delirium in ICU patients and is quick and easy to administer. It has been assessed as being reasonably accurate, with greater than 80% specificity, and is around 95% sensitive for diagnosis (when compared to the DSM-IV criteria) when applied to ICU patients (Gusmao-Flores et al., 2012). Additionally it shows good reliability between raters (Ely et al., 2001) and strong associations with clinical outcomes (Saczynski et al., 2012).

However it is not clear how well the CAM-ICU tool performs in the early post-operative period. The pathophysiology of Post Anesthesia Care Unit (PACU) delirium differs from delirium in other situations since it is largely driven by a few important causes such as central nervous system drug effects and the acute inflammatory response to surgery. In a sample of 91 elderly patients, Neufeld et al. (2013) found the CAM-ICU screening tool (completed about 30 min after waking) to miss a significant number of cases of true delirium with poor sensitivity (28%) but high specificity (98%) when compared to a formal psychiatric evaluation based on DSM criteria.

Additionally, from our clinical experience it is apparent that there are some further specific problems with categorization of delirium in the PACU using CAM-ICU criteria, namely,

1. It is possible to have evidence of disordered thinking and perceptual disturbances yet “pass” the CAM-ICU by completing the attention task correctly.
2. We also commonly observed that patients could demonstrate alternative clear cognitive or perceptual disturbances— mainly hallucinations, or an intermittent inability to speak, but still have passed the disordered thinking section of the CAM-ICU test.
3. Agitation is commonly associated with severe post-surgical pain; which could distract the patient and render them unable to attend to the CAM-ICU test.
4. Decreased level of arousal from residual anesthetic agent effects may increase the risk of inattention and result in a positive CAM-ICU screen for delirium—yet with the current interpretation of CAM-ICU it is not possible to subcategorize the etiology of hypoactive inattentiveness.

Given the unique clinical scenario of emergence from anesthesia and difficulties in diagnosing delirium in the PACU, we propose that the aspects of arousal, inattention, pain and disordered thinking contributing to delirium be considered. We therefore propose to modify and extend the CAM-ICU delirium screening tool to make it more suitable for patients in the immediate postoperative period; we have termed this the CAM-PACU score.

## Materials and Methods

This analysis is a sub-study of a larger ongoing study characterizing the clinical and EEG trajectories of patients emerging from general anesthesia (the ACCESS project^1^). Patients scheduled to undergo general anesthesia for surgery at the Waikato District Health Board Hospital, Hamilton, New Zealand, were recruited using a convenience sampling method. This observational study was approved by the New Zealand Health and Disability Ethics Committee (reference: 12/CEN/56), and all participants gave written informed consent in accordance with the Declaration of Helsinki.

Participants were interviewed by study staff at 15 min following arrival in the PACU for those patients who had responded to voice command in the operating theatre, and from return of responsiveness to command and expulsion of the airway for those patients transferred to PACU unresponsive with a laryngeal mask airway (LMA) *in situ*. Interviews were completed by two researchers (DFH and JDW) with clinical experience. At this time we asked participants to report their pain on a numerical rating scale of 0–10, and patient’s level of arousal was assessed using the Richmond Agitation and Sedation Scale (RASS; Ely et al., 2003). The RASS is a validated tool used to describe the level of arousal of patients on a 10 point scale. A score of zero reflects a calm alert state. Agitation is scored from +1 (anxious without aggressive or excessive movements) up to a maximum +4 (overtly combative, violent, danger to staff). Sedation is scored from −1 (drowsy but with sustained eye opening to voice) to −5 (unarousable to voice or physical stimulus).

The CAM-ICU was also administered at 15 min. The CAM-ICU test consists of RASS and two additional components; an attention test, and four logical questions. In the attention test, the patient is required to squeeze the hand of the investigator when the letter A is spoken in a list of 10 letters. Four A’s are spoken, and an incorrect response to two or more of the 4 A’s is designated as inattention. Then four simple logical questions to detect disordered thinking are presented. The four questions are “*Will a stone float on water?*”, “*Do fish swim in the sea?*”, “*Does one pound weigh more than two pounds?*” and “*Can you use a hammer to pound a nail?*”. If the patient makes more than one error in the logic questions then the criteria for disorganized thinking are met.

The diagnosis of delirium requires a patient to have evidence of inattention and to also either show a change in level of consciousness (as measured by the RASS, in our terminology equivalent to “arousal”), or show disordered thinking, in answering two or more of the four logic questions incorrectly. Hence, there are two pathways to a diagnosis of delirium with the CAM-ICU; inattention with a change in level of arousal, or inattention with signs of disordered thinking.

We also collected data on further manifestations of disordered cognition and perception namely:

i) visual and auditory hallucinations
ii) unreactive but eye-open state
iii) unable or unwilling to give verbal responses to questions or give a pain score

Any temporary confusion or disorientation immediately after arrival in the PACU (1–3 min) was ignored.

We used these criteria in addition to the CAM-ICU criteria to create an extended more inclusive evaluation, which we term the CAM-PACU. Also, if the patient showed signs of agitation (a RASS score above zero) but could report that they were in high pain, they were not considered as CAM-PACU positive.

Means between groups were compared using a one-way analysis of variance (ANOVA) test, and comparison of proportions was completed with a Chi-Square test. The level of significance for both was set at *p* = 0.05.

## Results

We analyzed 251 adult patients whose PACU data had been collected as part of another larger study. Three-hundred and five patients were enrolled into the study from the Waikato hospital, but 54 patients were excluded from this analysis due to recording failures associated with electroencephalographic (EEG) or patient monitoring recordings during emergence (27 patients), or due to PACU data (in its entirety) not being recorded (also 27 patients).

### Patient Demographics

Of the 251 patients included in the analysis, mean patient age was 59 years (SD 18 years), and 187 patients had sevoflurane as volatile anesthetic, the remaining 64 receiving desflurane. Mean length of operation was 111 min (SD 82 min), and the mean fentanyl-equivalent opioid effect-site concentration at end of surgery was 0.64 ng/ml (SD 0.57). The types of surgery patients underwent were general (40%), vascular (27%), urological (14%), or gynaecological surgery (12%). The remaining surgery types (7%) were primarily plastic surgery.

### Delirium

Thirty-three patients (14%) were diagnosed with delirium according to CAM-ICU criteria, but using our extended criteria an additional eleven patients showed signs of disordered thinking and received the CAM-PACU delirium designation (17%).

### Inattentive Delirium (CAM-ICU positive)

**Figure 1:**
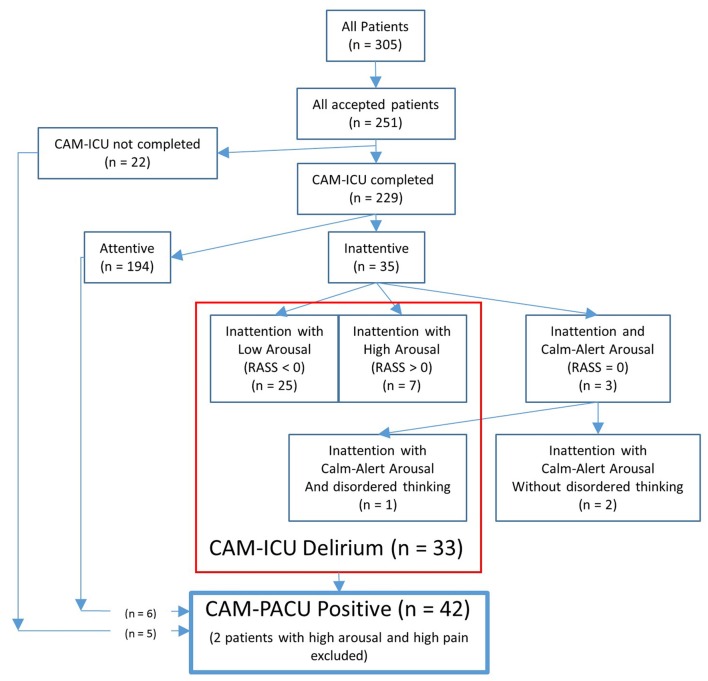
Flow-chart showing the number of patients diagnosed with delirium according to the confusion assessment method-intensive care unit (CAM-ICU), or who were confusion assessment method-post anesthesia care unit (CAM-PACU) positive.

Of the 229 patients who had had the CAM-ICU test completed, 35 patients (15%) failed the attention section of the CAM-ICU test (see Figure 1). Of these, three patients showed a calm and alert level of arousal (RASS = 0), and only one of these three patients failed the disorganized thinking section of the CAM-ICU and received the diagnosis of delirium. Of the remaining 32 patients showing inattention with altered levels of arousal (a RASS lower or higher than zero) thus screening positive for a motoric subtype of delirium, 25 showed inattention with low arousal (RASS <0, sub-diagnosed with hypoactive delirium), and seven showed inattention with high arousal (RASS >0, sub-diagnosed with hyperactive delirium). Patients with inattentive delirium had undergone longer operations (median operation length 116 vs. 87 min, *p* = 0.028).

### CAM-PACU

An additional six patients who had passed the CAM-ICU attention test, and five patients who had not received the CAM-ICU test, also showed signs of disordered thinking according to our CAM-PACU criteria. Of these 11 patients, five showed hallucinations (such as waving at no-one), three were in an unreactive eyes-open state, and three were unable/unwilling to express a pain score. Additionally, two patients showing inattention with high-arousal also reported high pain scores and were thus excluded from the CAM-PACU positive designation. A total of 42 patients were thus designated as CAM-PACU positive. Note also that of the 33 patients screening positive for delirium with the CAM-ICU, only one patient had delirium due to inattention with disordered thinking, but that 10 of these patients had also exhibited signs of disordered thinking according to our extended CAM-PACU criteria. Of these patients, four had hallucinations, three were in an unreactive state, and three were unable/unwilling to express a pain-score.

Patients categorized as CAM-PACU positive were older (median age 72 vs. 62 years, *p* = 0.010) and had undergone longer operations (median operation length 120 vs. 87 min, *p* = 0.003).

The proportion of patients diagnosed with CAM-PACU delirium was not statistically associated with the type of volatile anesthetic used during the operation (sevoflurane vs. desflurane, *p* > 0.05).

**Figure 2:**
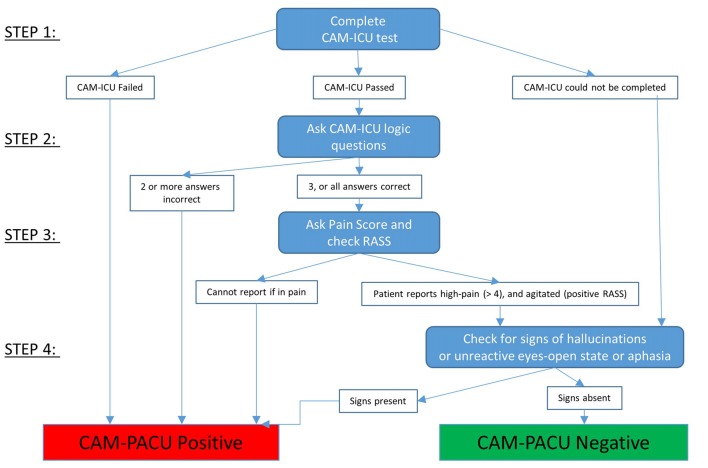
The four step process of using the CAM-PACU test for delirium and disordered thinking.

A flow diagram for using the CAM-PACU test is provided in Figure 2.

## Discussion

Thirty-three patients (14%) were diagnosed with delirium according to the CAM-ICU criteria, but using our extended criteria an additional eleven patients showed signs of disordered thinking.

With the addition of a few simple criteria the CAM-PACU captures patients with signs of disordered thinking that are not diagnosed with delirium using the CAM-ICU criteria. These were either patients who had passed the initial attention test (and were unable/unwilling to give verbal responses to questions), or who could not have the CAM-ICU test completed due to the constraints of the clinical situation, such as a patient having extreme nausea or sudden low blood-pressure, or using a bed-pan. While the group of patients diagnosed with delirium according to the CAM-ICU had undergone longer operations, this association was stronger with CAM-PACU criteria. In addition with CAM-PACU, an effect of increasing age also became statistically significant, showing that older patients are at particular risk of showing disordered thinking in the immediate postoperative period, the association between advancing age and delirium already being well-established (Saczynski et al., 2012). One issue is how the CAM-ICU is structured is that it gives precedent to firstly inattention, then to change in arousal, and only finally to disordered thinking. If some parts of CAM-ICU are “passed” the disorganized thinking element of evaluation is omitted.

Currently patients are discharged from PACU based on meeting criteria such as the Post-Anesthesia Recovery Scoring System (PARS) or the Aldrete Score. These scores require patients to be rousable on calling and able to move at least two extremities prior to discharge, but does not formally include any additional neurocognitive evaluation beyond this. As we predominantly observed the hypoactive subtype, it is likely these cases would go unnoticed without formal assessment. The European Society of Anaesthesiology now recommends screening of all patients before leaving the recovery room and continuing through the perioperative stay (Aldecoa et al., 2017). The CAM-ICU or CAM-PACU are relatively simple to do but are not yet a routine component of PACU care.

In developing the CAM-PACU, we potentially improve the sensitivity of CAM-ICU by including a patient group who have disordered thinking in the absence of inattention. Conversations with PACU nurses have led us to suspect that this subgroup of patients showing signs of disordered thinking may represent a different subset of patients than those simply showing inattention together with a change in (usually decreased) level of arousal. In this way we are attempting to focus in on one particular sub-component of delirium. A recent review by Guenther and Radtke (2011) noted that patients with sub-syndromal delirium, where not all criteria for delirium were met, had worse clinical outcomes than patients without signs of delirium (Skrobik et al., 2010). Some patients were unable to provide a pain score, verbally or non-verbally, despite the ability to respond to yes/no questions. Other clinical observations included reduced eye contact, sharing of bizarre information, and the need for repetition of instructions. These behaviors are consistent with aphasia and cognitive-communication disorders, but an assessment of language and communicative function would be required to make such claims.

In this study, we have chosen to retain all patients diagnosed with delirium due to inattention with altered levels of consciousness (or arousal) as CAM-PACU positive. Yet there is some disagreement in the literature as to the significance of low arousal in diagnosing delirium, as commonly occurs in the PACU. In an interpretive guide to the DSM5 criteria (European Delirium Association, 2014) the European and American Delirium Associations recommended that only cases of coma, i.e., a severe lack of arousal, be excluded from delirium diagnosis as both decreased attention and arousal contribute to delirium. The hypoactive subtype of delirium (Meagher et al., 2012) presents special problems for diagnosis in PACU; distinguishing hypoactive delirium from residual sedation can be a question of interpretation (Haenggi et al., 2013; Winter et al., 2015). As shown by Card et al. (2015), delirium as assessed by inattention with decreased arousal is common immediately after waking from general anesthesia (31%), but incidence rates decrease at an exponential rate during PACU stay, which is suggestive of a key role of anesthetic clearance. They name this phenomenon a “rapidly reversible sedative-related delirium”, and is analogous to the transient abnormal neurological signs that show in healthy patients during emergence from anesthesia (Rosenberg et al., 1981). If delirium is defined as a reduced level of consciousness with inattention, then a large proportion of otherwise healthy patients show delirium at some point while waking from general anesthesia, i.e., for the PACU situation this terminology is perhaps *too* inclusive.

To exactly what degree residual sedation contributes to the diagnosis of delirium, and how this effect could be determined remains unclear. The gold standard of delirium diagnosis is according to the DSM5 criteria. These criteria are not easily applied to the post-operative situation (while sedation is waning) and may lead to inaccuracies regarding the incidence of PACU delirium (Winter et al., 2015). In addition to residual sedation from incomplete anesthetic clearance, diagnosis of delirium in the PACU is also potentially confounded by the presence of residual opioids, the possibility of incomplete emergence from anesthesia, and surgical insult, but this area of research is quite under-developed. While the etiology of PACU delirium may differ somewhat than that usually encountered, the pathophysiology of non-PACU delirium itself is complex and multifactorial (Maldonado, 2008). To our knowledge, no detailed phenomenological analysis of the early post-anesthesia recovery period has been completed, but an upcoming study by Maier et al. (2017) should help shed light on the sequences and timing of the recovery of various neurocognitive domains in healthy young volunteers.

Our clinical observations indicate that some patients may have been experiencing difficulty with language and communication. Accordingly, the inclusion of an informal assessment of language and communicative function may yield valuable information in future studies. For example, aphasia is present in to 38% of patients with left-hemisphere stroke (Pedersen et al., 1995), and cognitive-communication deficits are present in 50%–78% of patients with right-hemisphere stroke (Benton and Bryan, 1996; Blake et al., 2002; Hewetson et al., 2017). It is possible that the temporary occurrence of deficits in language and communicative function in the PACU may be a warning of current or future neurovascular compromise. Importantly, this could provide a unique opportunity for early intervention and/or rehabilitation strategies to improve outcomes.

A number of additional limitations apply to this study. We have not performed a formal DSM-5 comparison. While this test is considered the gold standard for delirium, it is not designed to be applied in the postsurgical setting. However, the features assessed in the CAM do broadly align with DSM-5 criteria, although notably DSM-V requires a disturbance in awareness (reduced orientation to the environment), and disorientation is not formally assessed in the CAM-ICU work sheet. In this study we found associations between age and PACU delirium and length of surgery and PACU-delirium, which are consistent with the existing literature on post-operative delirium. Although emotional (depressive) and cognitive impairments may increase the likelihood of a patient developing delirium, we did not conduct any pre-operative neurocognitive evaluations or depression scoring, which may have provided additional useful information regarding delirium risk factors.

Also, in this study we do not have any further measures of clinical impact of this particular subgroup, which will need to be teased out in further studies. There is a need for further large prospective observational studies to determine which features of PACU delirium are associated with further episodes of delirium and poor outcomes.

## Author Contributions

PG and JS designed and oversaw the study. DH and JW completed recordings. DH, JS, JW, AR and AG analyzed data and wrote the manuscript. All authors interpreted data and made critical revisions to the final manuscript.

## Conflict of Interest Statement

The authors declare that the research was conducted in the absence of any commercial or financial relationships that could be construed as a potential conflict of interest.

## Footnotes

^1^www.accesshq.org
